# Dermanyssosis in the Urban Context: When the One Health Paradigm Is Put into Practice

**DOI:** 10.3390/pathogens11121396

**Published:** 2022-11-23

**Authors:** Alessandra Barlaam, Antonella Puccini, Maria Filomena Caiaffa, Danilo Di Bona, Luigi Macchia, Annunziata Giangaspero

**Affiliations:** 1 Department of Agriculture, Food, Natural Resources and Engineering (DAFNE), University of Foggia, 71121 Foggia, Italy; 2 Agenzia Sanitaria Locale, 71122 Foggia, Italy; 3 Department of Medical and Surgical Sciences, School and Chair of Allergology and Clinical Immunology, University of Foggia, 71122 Foggia, Italy; 4 Department of Emergency and Organ Transplantation, School and Chair of Allergology and Clinical Immunology, University of Bari-Aldo Moro, 70121 Bari, Italy

**Keywords:** poultry red mite, pigeons, human dermatitis, one health paradigm, Italy

## Abstract

Poultry red mites (*Dermanyssus gallinae*) are primarily ectoparasites of laying hens but also parasitize synanthropic birds in urban contexts. This mite can occasionally attack mammals, including humans, and cause mild to severe dermatitis. Attacks by zoonotic Mesostigmata mites are currently an increasing but still neglected problem of urban life. The authors present two cases of dermanyssosis involving two health workers at a hospital, linked to air conditioning outdoor units colonized by pigeons. Videos that describe the environmental contamination by *D. gallinae* and show where the infestation originated are presented. In addition, the authors update the literature of all urban cases, which, to date, reports over 240 clinical cases, mostly in private homes but also in public buildings. Dermatitis due to these mites is often unrecognized and, therefore, misdiagnosed. This report describes how the two cases herein reported were rapidly resolved thanks to the close cooperation between veterinary parasitologists and allergologists. It is crucial to raise awareness of the problem among general practitioners and specialists. In addition, the authors suggest a reconsideration of urban architectural choices that increase the public health risk posed by dermanyssosis and other diseases related to synanthropic birds.

## 1. Introduction

The poultry red mite *Dermanyssus gallinae* (De Geer 1778) (Acari: Dermanyssidae) is a tiny (0.5–1 mm long) nocturnal non-burrowing hematophagous zoonotic ectoparasite. *Dermanyssus gallinae sensu stricto* is a major pest of poultry farms worldwide that primarily targets laying hens [1] and is responsible for economic losses of over EUR 230 million per year in Europe, because of its impact on animal welfare and health. It leads to stress, feather-pecking, increased self-grooming and cannibalism, reduced egg production and quality and, sometimes, mortality due to anemia [2]. In addition, *D. gallinae* may carry a number of zoonotic pathogens both viral, e.g., influenza type A viruses, and bacterial, e.g., *Salmonella enteritidis*, *Erysipelothrix rhusiopathiae*, *Chlamydia psittaci*, *Escherichia coli*, *Pasteurella multocida*, *Coxiella burnetii* and *Listeria monocytogenes*. Its vectorial role for some of these has been demonstrated [3].

In the urban context, *D. gallinae Lineage 1* poses a threat to synanthropic birds (e.g., pigeons, sparrows, starlings, doves) [4], and, as in poultry farms, it can occasionally attack humans.

The mite’s life cycle consists of egg, larva (non-feeding stage), protonymph, deutonymph and male and female adult (feeding stages) and lasts an average of two weeks. However, at high temperatures (35 °C) and with relative humidity above 70%, the life cycle can be completed in only one week [5,6].

Dermanyssosis is considered an occupational disease in the poultry industry [7]. In fact, the mites attack humans when the infestation level is high and farmers, farm workers or visitors are not adequately protected. In the urban context, *D. gallinae* can infest humans when synanthropic bird nests are in close proximity to residential and public buildings on balconies, attics or behind heating/air-conditioning outdoor units. When the chicks fledge, birds usually abandon the nest and the mites, in the absence of their preferential host, look for alternative hosts, including humans. Mites can easily pass through windows at any time of the day, mostly in late spring-early summer and autumn. Human victims present a scabies-like dermatitis with erythematous maculopapular or papulovesicular lesions, erythematous rashes, small papules and blisters, urticarial plaques and erythema, which sometimes displays a visible central puncture mark, urticarioid manifestations and severe itchiness [8]. These lesions are commonly reported on various parts of the body, including the arms, hands, chest and legs, although lesions to the auditory meatus [9] and scalp have also been documented [10].

Human attacks have mostly been recorded during daylight hours in workplaces or at night in private houses. Symptoms can persist for a long time in the absence of a correct diagnosis.

The authors present two cases of dermanyssosis in two health workers at a hospital, where the infestation was linked to air conditioning outdoor units colonized by pigeons. This report highlights that prompt intervention to investigate the origin of the dermatitis and the allergologist in charge of the case’s awareness of the dermanyssosis enabled a rapid resolution of the problem. In addition, the authors update the literature regarding all the urban cases of human dermanyssosis in Europe recorded to date.

## 2. Case Presentation

Two female health workers in their shared office at the University Hospital of Bari, Italy, were infested by *D. gallinae*. Patient 1, aged 64, and patient 2, aged 24, were referred to the Allergology Unit of the same hospital.

**Figure 1:**
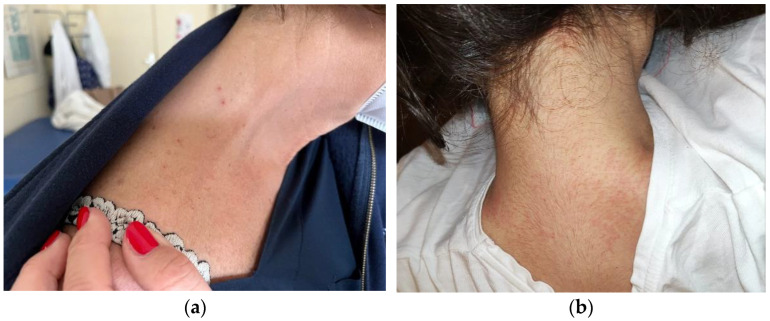
*Dermanyssus* provoked multiple, reddish and itchy papules. (**a**) Patient 1, base of the neck; (**b**) Patient 2, back of the neck.

At clinical examination, the patients presented dermatitis with numerous erythematous papules and urticaria-like lesions. Patient 1 presented lesions on the neck, abdomen and legs. Patient 2 presented lesions on the neck and nape (Figure 1a,b). Both patients reported that the lesions were itchy and that they had suffered intense itchiness for the past two days, during daytime at work and nighttime at home.

Neither of the patients had a history of contact with animals. Moreover, no other members of their households showed similar symptoms.

The patients reported seeing tiny creatures on their desks and computers while working. Therefore, the allergologist in charge, aware of the existence of human dermanyssosis, requested that the patients’ office be inspected by parasitologists to track down the possible source of the infestation. During the inspection, crawling mites were found on the computer desk and screen (Supplementary Material, Video S1), windowsills and window frames. Moreover, abandoned pigeon nests were found outside the window, between the air conditioning outdoor unit and the outer wall of the building, in close proximity to the window of the infested room (Supplementary Material, Video S2).

In both patients, allergic contact dermatitis was ruled out by patch testing for common haptens, which proved negative.

Mites were collected using a small damp brush and transported alive to the parasitology laboratory of the Department of Agriculture, Food, Natural Resources and Engineering (DAFNE), University of Foggia (Italy). In the laboratory, the mites were macerated in lactophenol for one week at 45 °C on a hot plate and then mounted on slides with Hoyer’s medium, for light microscopy observations (Axio Zeiss Imager A1, ZEISS, Oberkochen, Germany). The specimens were identified as *Dermanyssus gallinae*, according to the morphological characters described in Di Palma et al. [11]. It was suggested to have the empty nests removed and an environmental disinfestation carried out by a specialist company. Following the nests removal, the room’s air-conditioning outdoor unit was protected with additional pigeon deterrents to prevent further nesting and the workplace was subjected to two cycles of fumigation with pyrethroids; no access was allowed for 48 h. Once these procedures had been completed, the two patients returned to work in their office and no further mite attacks were reported. Complete regression of their symptoms occurred in 7–8 days.

## 3. Discussion

Dermatitis caused by zoonotic Mesostigmata mites is currently an increasing but still neglected problem in urban settings. It extends far beyond the Mediterranean area and has also been reported in some northern European countries.

After the reports listed by Cafiero et al. [12], another eight urban cases of dermanyssosis in humans in Europe have been recorded, i.e., two cases in France [13], one case in Greece [14], two cases in Hungary [15] and three in Italy [16]. To date, over 240 clinical cases of urban infestations caused by *D. gallinae* have been reported. The vast majority occurs in private homes/apartments (over 187), followed by offices/public buildings (over 30 clinical cases), hospitals (over 10 clinical cases) and unknown sites (3 clinical cases) (Table 1).

In general, the number of cases/outbreaks is greatly underestimated since the lesions are uncharacteristic and *D. gallinae* bites may be confused with lesions due to scabies (by *Sarcoptes scabiei*), cheyletiellosis (by *Cheyletiella*), trombiculosis (by *Trombicula*), bedbug infestation (by *Cimex lectularius*) or with urticarial dermatitis [12]. They may also be ascribed to allergies or confused with delusional ectoparasitosis [50].

There is often a lack of understanding around dermatitis owing its origin to mites, with this diagnosis only suspected or delayed due to a lack of awareness by clinicians. Allergologists and dermatologists should always recommend the inspection of potential sites in order to detect, collect and correctly identify the mites. Such inspections should be carried out by expert parasitologists. In fact, *D. gallinae* may be mistaken with other avian mites such as the ones belonging to the *Ornithonyssus* genus (Acari: Macronyssidae), which are also responsible for human dermatitis [15,48], but have a different morphology, biology and relationship with the host. When the cause of infestation goes unrecognized by physicians, the treatments administered to relieve symptoms, mainly antihistamines and steroids, are only effective in the short term; however, the clinical signs usually recur when the treatments end. Antiparasitic shampoos, antibiotics or even tranquilizers are unnecessary to treat against or prevent the reoccurrence of *D. gallinae*. This aspect is crucial given that *Dermanyssus* may be a vector/reservoir of certain zoonotic agents, such as *Borrelia burgdorferi s.l.* and *Coxiella burnetii,* detected in *D. gallinae* during two outbreaks of human dermatitis [51]. Another urban outbreak involved *Dermanyssus* spp. mites, from which *Bartonella quintana* DNA was detected [37].

In the cases herein described, the awareness of dermanyssosis by clinicians enabled a prompt intervention by the parasitologists, the correct identification of the mites and a rapid resolution of the problem.

## 4. Conclusions

In conclusion, it is well known that several synanthropic bird species colonize our cities and towns and that the most successful ones are the pigeons (*Columba livia*), which may harbor several organisms pathogenic for humans, including *D. gallinae*. Pigeons are responsible for over 90% of the clinical cases of dermanyssosis in urban settings [14].

**Figure 2:**
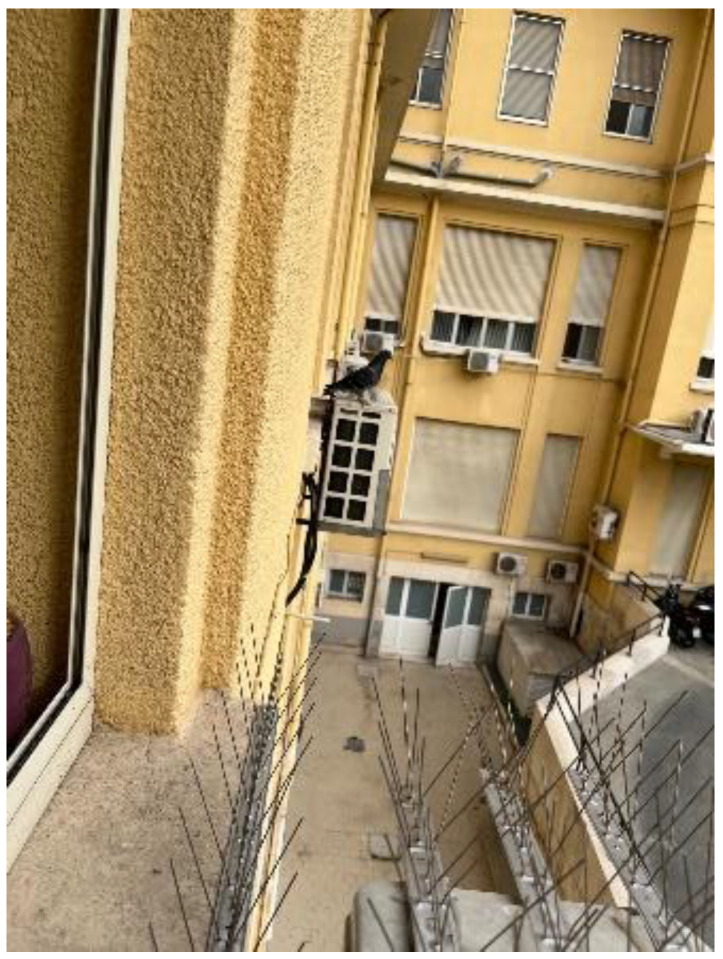
Several external air conditioning units installed under the windows of public offices.

Our experience confirms that dermanyssosis outbreaks are common, particularly when there are incorrect or unsuitable structural interventions (e.g., crevices and niches on facades and roofs). Air conditioning external units can also contribute to the problem; in fact, they provide pigeons with an excellent place to build their nests (Figure 2).

Given the potential for *D. gallinae* infestation in urban areas, it is no longer possible to delay a radical re-consideration of urban architectural choices. It becomes a matter of urgency to implement targeted regulations and to encourage communication between urban planners and public health operators. The problem can no longer be ignored and these measures must be implemented in order to safeguard public health in urban contexts.

## Figures and Tables

**Table 1:** Reports of urban cases of dermanyssosis on humans in Europe (1930–2022).

City/Region, Country	Year of Record	Location Number of People Involved				Source	References
		Private Homes/Apartments	Hospital	Offices	Unknown		
Zurich, Switzerland	1930	NR				Synanthropic birds	[17]
Rotterdam, The Netherlands	1961	23				Pigeons, tiger finches, parakeets, canaries	[18]
Hamburg, Germany	1967	12				Ventilation openings infested by mites from bird nest	[19]
Cornwall, UK	1967 and 1968	4				Starlings’ nest found in crevice where waste pipe exits the house (1967); Nesting material found in loft close to girl’s bedroom (1968)	[20]
Danzig, Poland	1967		NR			Pigeon nest	[21]
London, UK	1969		NR			Abandoned pigeon nest	[22]
Danzig, Poland	1971			NR		Pigeon nests	[23]
Kielce, Poland	1974			1		Immediately following removal of pigeon nest close to office	[24]
Vienna, Austria	1981	4		4		Pigeon nest outside a factory window	[25]
UK	1985	4	4			Infested pigeons nesting on window ledges of two hospital wards	[26]
Basel, Switzerland	1988			1		Laboratory infestation by *D. gallinae,* escaped from plastic bag containing pigeon feces harboring red mites	[27]
Nijmegen region, Netherlands	1996	3				Bird nests under roof tiles	[28]
Apulia, Basilicata, and Campania regions, Italy	2001–2007	6		17		Abandoned feral pigeon nests near infested buildings (hole in the wall/behind air-conditioning units)	[29,30]
Amsterdam, Netherlands	2003–2008	96				Synanthropic bird nests, mainly pigeons, in outer walls of apartments	[31]
Tilburg region, Netherlands	2005	NR				Pigeon nest with dead pigeon adjacent to the bedroom window of the patient	[32]
Modena, Italy	2005 and 2006	1		NR		Feral pigeons roosting and nesting on school roof; Colony of pigeons nesting on chimney pot of attic	[33]
Valencia, Bétera-Camp de Turia, Spain	2006–2008	>3		NR		Abandoned nest close to cottage/pigeons’ nest behind air-conditioning units	[34]
Modena, Italy	2007				3	Removal of pigeon nests from window ledge	[35]
Apulia region, Italy	2007	4				Abandoned pigeon nest under gutter between balconies of two apartments	[30,36]
Czech Republic	2007	4				Mites entered apartment via hole in the roof	[37]
Créteil, France	2008		1			Abandoned pigeon nest near a window	[38]
Apulia and Basilicata regions, Italy	2008 and 2009	9				Abandoned pigeon nests (2008); Sparrow nest (2009)	[39]
Kütahya, Turkey	2009	>1				Mites from pigeons through air ventilation system	[40]
Basel, Switzerland	2009	NR				Feral pigeons using balcony for roosting/breeding	[41]
Puglia, Italy	2010		>4			Feral pigeons using window ledge near the infested rooms for roosting/breeding	[42]
Apulia, Italy	2011	3				Following removal of an active pigeon nest close to the house	[43]
Barcelona, Spain	2013	3				Pigeon nests near balcony	[44]
Pančevo, Serbia	2008 and 2011	5				Mites in nearby dove nest entering apartment through cracks in frame of balcony door	[45]
Apulia, Italy	2015			1		Abandoned pigeon nests in proximity to air-conditioner external unit	[46]
Apulia and Molise regions, Italy	2013 and 2015–2017	6		1		Abandoned pigeon nests (2013, 2015–2017); Caged canaries (2015)	[47,48]
Ferrara, Italy	2015			4		Colony of feral pigeons located on the roof of the building	[49]
Utrecht, Netherlands	2016	1				Pigeon nest	[12]
Paris, France	2020	1				Pigeon nest behind the windows of the office	[13]
				1		Pigeon nest on the flat terrace	
Argostoli, Kefalonia, Greece	2021	1				Swallow nest	[14]
Hungary, Budapest	2021	2				Pigeon nest attached to the outside wall under eaves	[15]
Catania, Italy	2021	1				NR	[16]
Apulia regionBari, Italy	2022			2		Abandoned feral pigeon nest under the office window between the building wall and air-conditioning unit	Current contribution
TOTAL		>187 cases	>10	>30	3		

NR: Not reported.

## Data Availability

Not applicable.

## Supplementary Materials

The following supporting information can be downloaded at: https://www.mdpi.com/article/10.3390/pathogens11121396/s1. Video S1: Crawling mites in the office environment, on the computer desk. Video S2: Identification of the infestation source: abandoned pigeon nests can be seen near infested rooms, just outside the windows, and between the air conditioning unit and the outer wall of the building.
